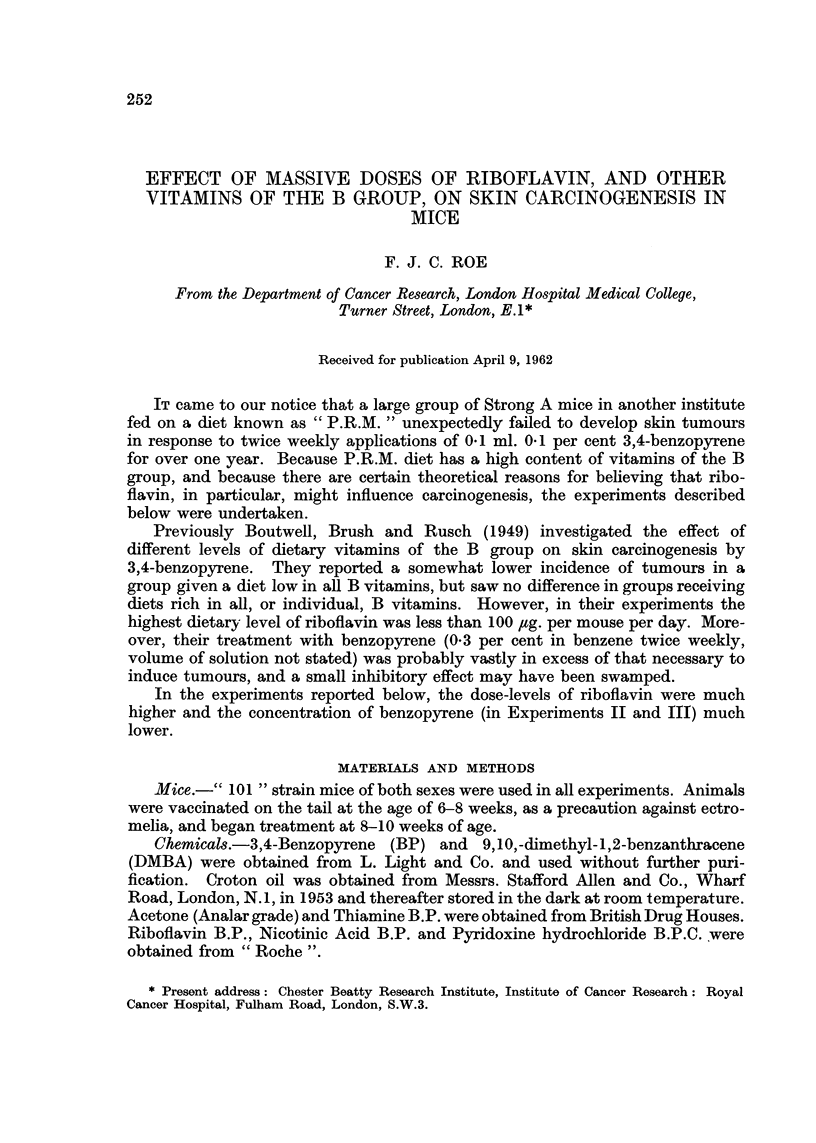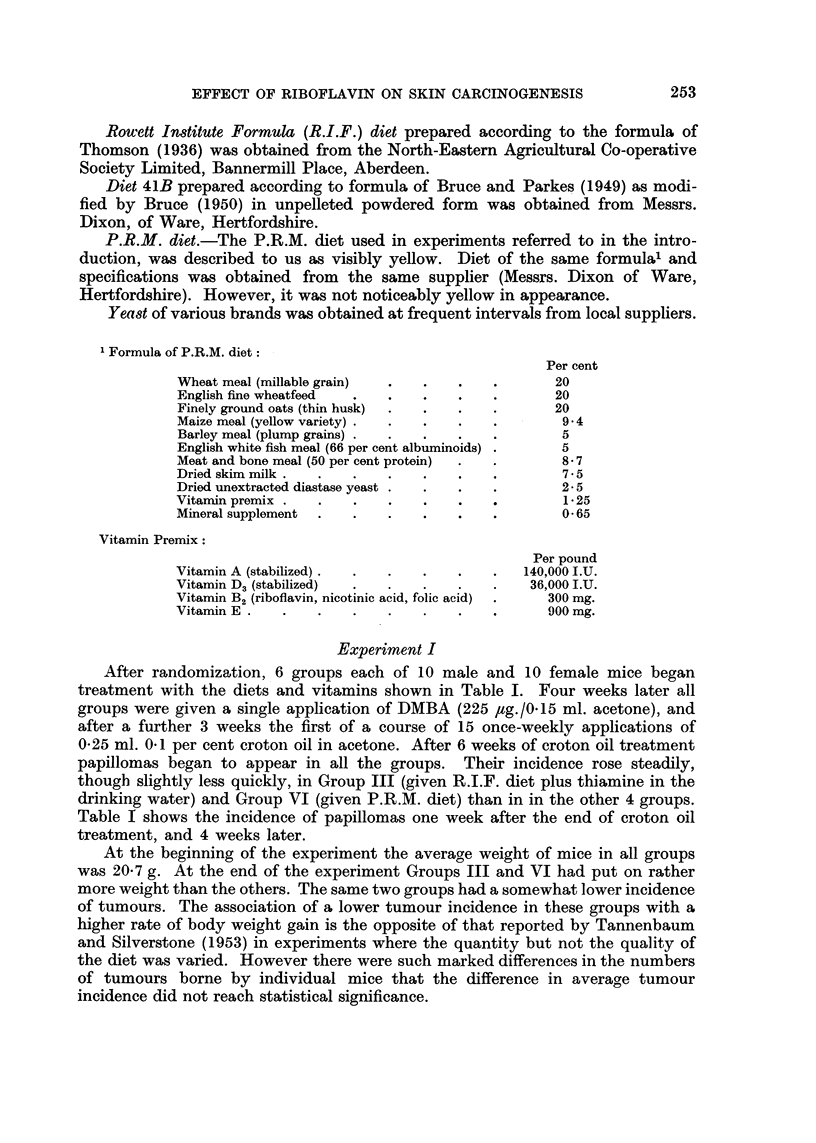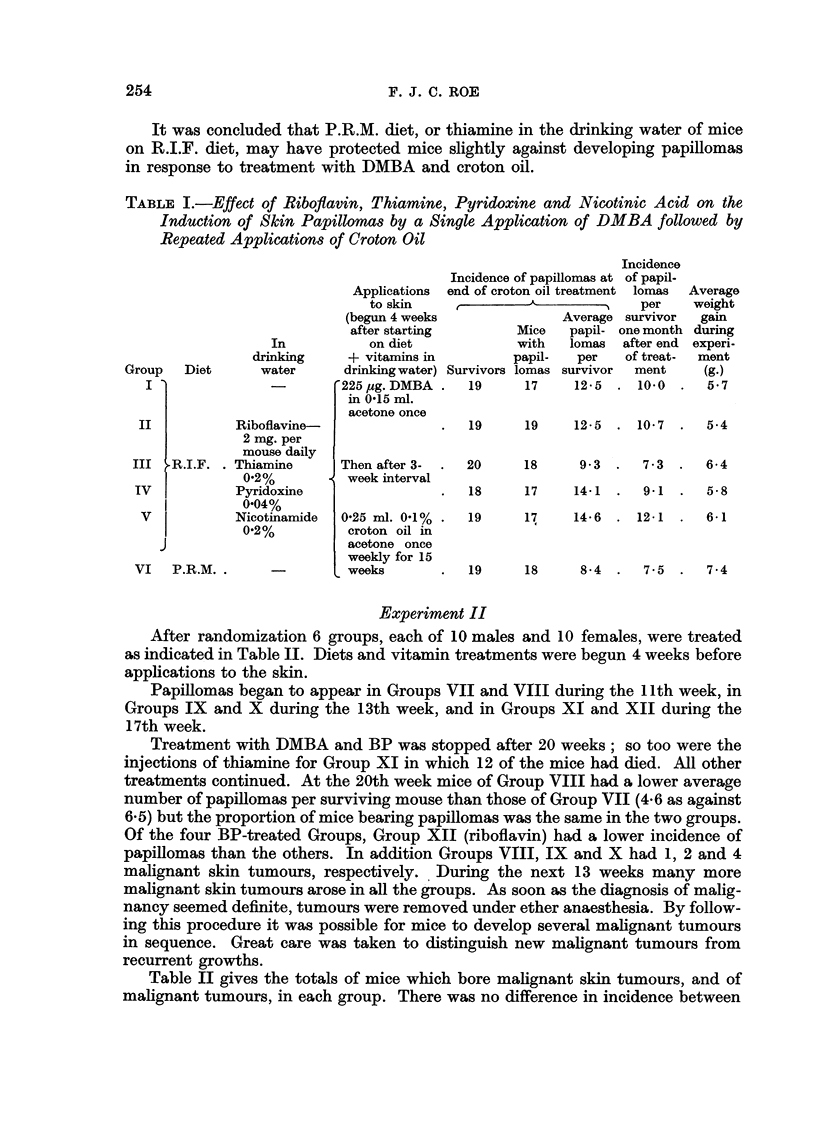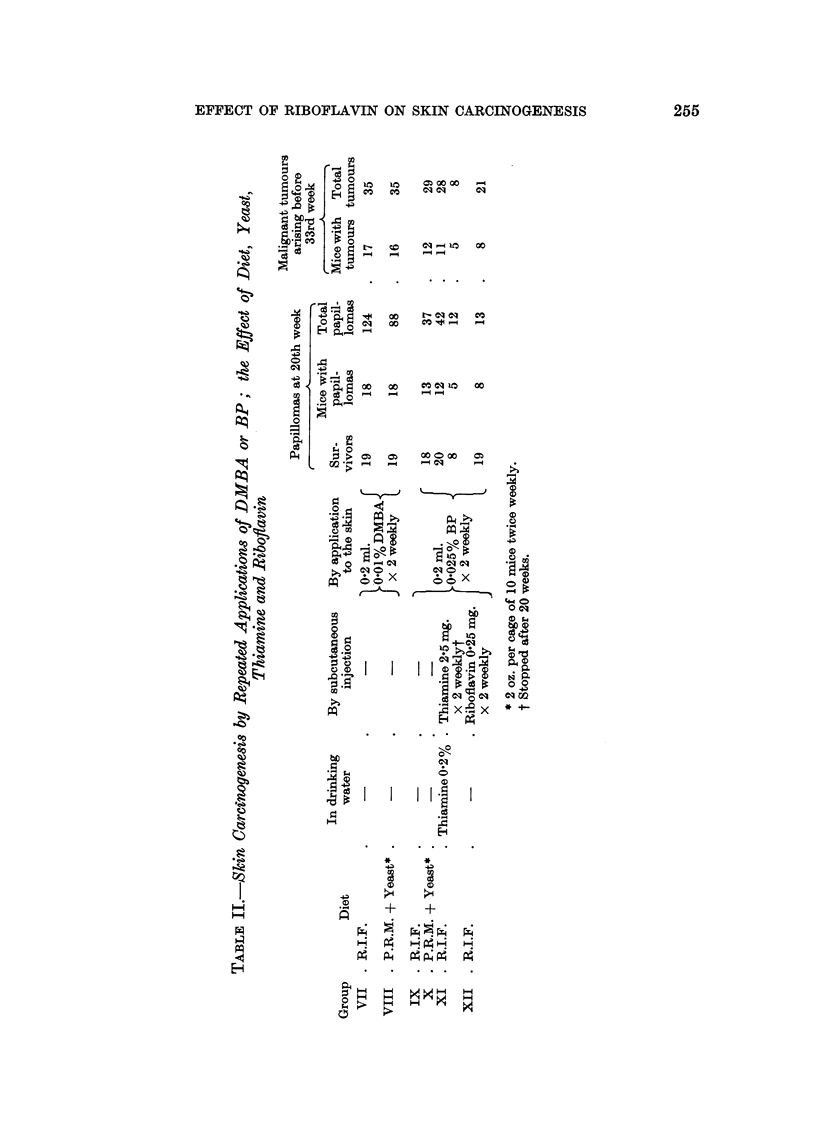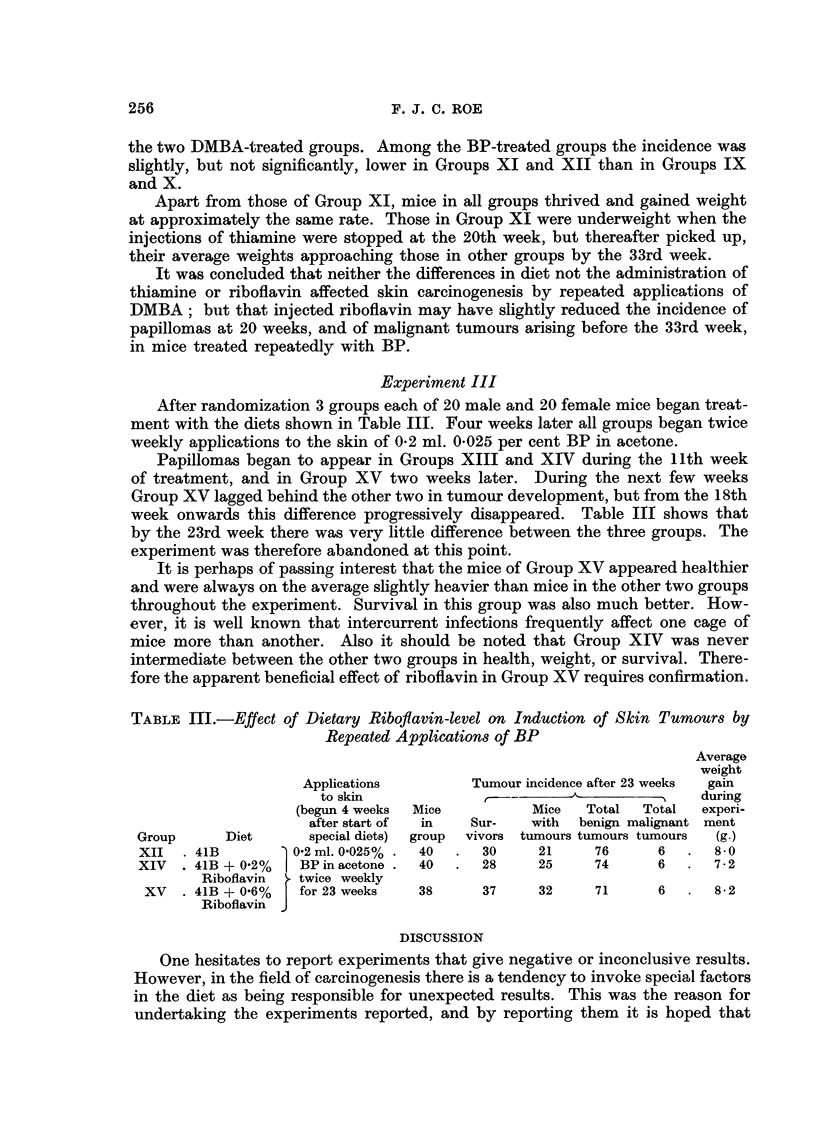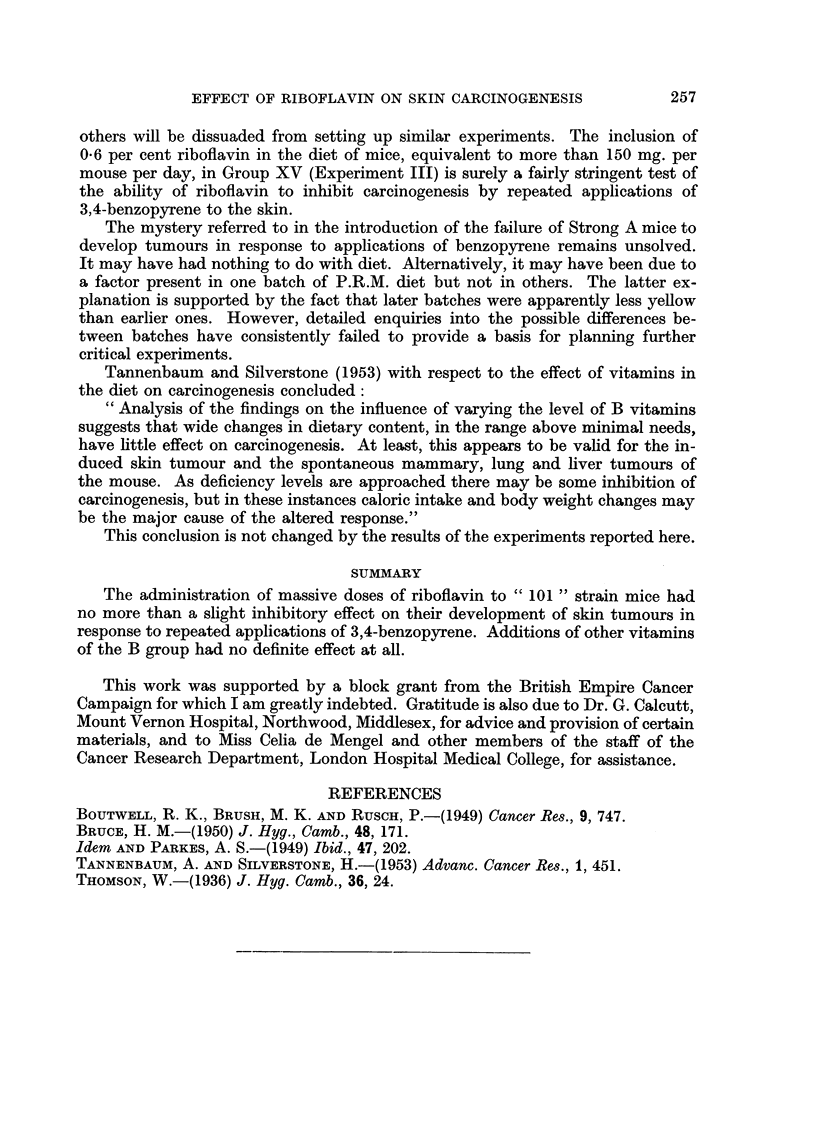# Effect of Massive Doses of Riboflavin, and Other Vitamins of the B Group, on Skin Carcinogenesis in Mice

**DOI:** 10.1038/bjc.1962.29

**Published:** 1962-06

**Authors:** F. J. C. Roe


### EFFECT OF MASSIVE DOSES OF RIBOFLAVIN, AND OTHER VITAMINS OF THE B GROUP, ON SKIN CARCINOGENESIS IN MICE

F. J. C. ROE

*From the Department of Cancer Research, London Hospital Medical College, Turner Street, London, E.1**

Received for publication April 9, 1962

It came to our notice that a large group of Strong A mice in another institute fed on a diet known as " P.R.M. " unexpectedly failed to develop skin tumours in response to twice weekly applications of 0·1 ml. 0·1 per cent 3,4-benzopyrene for over one year. Because P.R.M. diet has a high content of vitamins of the B group, and because there are certain theoretical reasons for believing that riboflavin, in particular, might influence carcinogenesis, the experiments described below were undertaken.

Previously Boutwell, Brush and Rusch (1949) investigated the effect of different levels of dietary vitamins of the B group on skin carcinogenesis by 3,4-benzopyrene. They reported a somewhat lower incidence of tumours in a group given a diet low in all B vitamins, but saw no difference in groups receiving diets rich in all, or individual, B vitamins. However, in their experiments the highest dietary level of riboflavin was less than 100 $\mu$g. per mouse per day. Moreover, their treatment with benzopyrene (0·3 per cent in benzene twice weekly, volume of solution not stated) was probably vastly in excess of that necessary to induce tumours, and a small inhibitory effect may have been swamped.

In the experiments reported below, the dose-levels of riboflavin were much higher and the concentration of benzopyrene (in Experiments II and III) much lower.

### MATERIALS AND METHODS

*Mice.*—" 101 " strain mice of both sexes were used in all experiments. Animals were vaccinated on the tail at the age of 6–8 weeks, as a precaution against ectromelia, and began treatment at 8–10 weeks of age.

*Chemicals.*—3,4-Benzopyrene (BP) and 9,10,-dimethyl-1,2-benzanthracene (DMBA) were obtained from L. Light and Co. and used without further purification. Croton oil was obtained from Messrs. Stafford Allen and Co., Wharf Road, London, N.1, in 1953 and thereafter stored in the dark at room temperature. Acetone (Analar grade) and Thiamine B.P. were obtained from British Drug Houses. Riboflavin B.P., Nicotinic Acid B.P. and Pyridoxine hydrochloride B.P.C. were obtained from " Roche ".

* Present address: Chester Beatty Research Institute, Institute of Cancer Research: Royal Cancer Hospital, Fulham Road, London, S.W.3.

*Rowett Institute Formula (R.I.F.) diet* prepared according to the formula of Thomson (1936) was obtained from the North-Eastern Agricultural Co-operative Society Limited, Bannermill Place, Aberdeen.

*Diet 41B* prepared according to formula of Bruce and Parkes (1949) as modified by Bruce (1950) in unpelleted powdered form was obtained from Messrs. Dixon, of Ware, Hertfordshire.

*P.R.M. diet.*—The P.R.M. diet used in experiments referred to in the introduction, was described to us as visibly yellow. Diet of the same formula[1] and specifications was obtained from the same supplier (Messrs. Dixon of Ware, Hertfordshire). However, it was not noticeably yellow in appearance.

*Yeast* of various brands was obtained at frequent intervals from local suppliers.

[1] Formula of P.R.M. diet:

| | Per cent |
|---|---|
| Wheat meal (millable grain) | 20 |
| English fine wheatfeed | 20 |
| Finely ground oats (thin husk) | 20 |
| Maize meal (yellow variety) | 9·4 |
| Barley meal (plump grains) | 5 |
| English white fish meal (66 per cent albuminoids) | 5 |
| Meat and bone meal (50 per cent protein) | 8·7 |
| Dried skim milk | 7·5 |
| Dried unextracted diastase yeast | 2·5 |
| Vitamin premix | 1·25 |
| Mineral supplement | 0·65 |

Vitamin Premix:

| | Per pound |
|---|---|
| Vitamin A (stabilized) | 140,000 I.U. |
| Vitamin $D_3$ (stabilized) | 36,000 I.U. |
| Vitamin $B_2$ (riboflavin, nicotinic acid, folic acid) | 300 mg. |
| Vitamin E | 900 mg. |

### *Experiment I*

After randomization, 6 groups each of 10 male and 10 female mice began treatment with the diets and vitamins shown in Table I. Four weeks later all groups were given a single application of DMBA (225 $\mu$g./0·15 ml. acetone), and after a further 3 weeks the first of a course of 15 once-weekly applications of 0·25 ml. 0·1 per cent croton oil in acetone. After 6 weeks of croton oil treatment papillomas began to appear in all the groups. Their incidence rose steadily, though slightly less quickly, in Group III (given R.I.F. diet plus thiamine in the drinking water) and Group VI (given P.R.M. diet) than in in the other 4 groups. Table I shows the incidence of papillomas one week after the end of croton oil treatment, and 4 weeks later.

At the beginning of the experiment the average weight of mice in all groups was 20·7 g. At the end of the experiment Groups III and VI had put on rather more weight than the others. The same two groups had a somewhat lower incidence of tumours. The association of a lower tumour incidence in these groups with a higher rate of body weight gain is the opposite of that reported by Tannenbaum and Silverstone (1953) in experiments where the quantity but not the quality of the diet was varied. However there were such marked differences in the numbers of tumours borne by individual mice that the difference in average tumour incidence did not reach statistical significance.

It was concluded that P.R.M. diet, or thiamine in the drinking water of mice on R.I.F. diet, may have protected mice slightly against developing papillomas in response to treatment with DMBA and croton oil.

TABLE I.—*Effect of Riboflavin, Thiamine, Pyridoxine and Nicotinic Acid on the Induction of Skin Papillomas by a Single Application of DMBA followed by Repeated Applications of Croton Oil*

| Group | Diet | In drinking water | Applications to skin (begun 4 weeks after starting on diet + vitamins in drinking water) | Incidence of papillomas at end of croton oil treatment: Survivors | Mice with papillomas | Average papillomas per survivor | Incidence of papillomas per survivor one month after end of treatment | Average weight gain during experiment (g.) |
|---|---|---|---|---|---|---|---|---|
| I | R.I.F. | — | 225 μg. DMBA in 0·15 ml. acetone once | 19 | 17 | 12·5 | 10·0 | 5·7 |
| II | R.I.F. | Riboflavine—2 mg. per mouse daily | | 19 | 19 | 12·5 | 10·7 | 5·4 |
| III | R.I.F. | Thiamine 0·2% | Then after 3-week interval | 20 | 18 | 9·3 | 7·3 | 6·4 |
| IV | R.I.F. | Pyridoxine 0·04% | | 18 | 17 | 14·1 | 9·1 | 5·8 |
| V | R.I.F. | Nicotinamide 0·2% | 0·25 ml. 0·1% croton oil in acetone once weekly for 15 weeks | 19 | 17 | 14·6 | 12·1 | 6·1 |
| VI | P.R.M. | — | | 19 | 18 | 8·4 | 7·5 | 7·4 |

*Experiment II*

After randomization 6 groups, each of 10 males and 10 females, were treated as indicated in Table II. Diets and vitamin treatments were begun 4 weeks before applications to the skin.

Papillomas began to appear in Groups VII and VIII during the 11th week, in Groups IX and X during the 13th week, and in Groups XI and XII during the 17th week.

Treatment with DMBA and BP was stopped after 20 weeks ; so too were the injections of thiamine for Group XI in which 12 of the mice had died. All other treatments continued. At the 20th week mice of Group VIII had a lower average number of papillomas per surviving mouse than those of Group VII (4·6 as against 6·5) but the proportion of mice bearing papillomas was the same in the two groups. Of the four BP-treated Groups, Group XII (riboflavin) had a lower incidence of papillomas than the others. In addition Groups VIII, IX and X had 1, 2 and 4 malignant skin tumours, respectively. During the next 13 weeks many more malignant skin tumours arose in all the groups. As soon as the diagnosis of malignancy seemed definite, tumours were removed under ether anaesthesia. By following this procedure it was possible for mice to develop several malignant tumours in sequence. Great care was taken to distinguish new malignant tumours from recurrent growths.

Table II gives the totals of mice which bore malignant skin tumours, and of malignant tumours, in each group. There was no difference in incidence between

TABLE II.—*Skin Carcinogenesis by Repeated Applications of DMBA or BP; the Effect of Diet, Yeast, Thiamine and Riboflavin*

| Group | Diet | In drinking water | By subcutaneous injection | By application to the skin | Papillomas at 20th week: Survivors | Papillomas at 20th week: Mice with papillomas | Papillomas at 20th week: Total papillomas | Malignant tumours arising before 33rd week: Mice with tumours | Malignant tumours arising before 33rd week: Total tumours |
|---|---|---|---|---|---|---|---|---|---|
| VII | R.I.F. | — | — | 0·2 ml. 0·01% DMBA × 2 weekly | 19 | 18 | 124 | 17 | 35 |
| VIII | P.R.M. + Yeast* | — | — | (as above) | 19 | 18 | 88 | 16 | 35 |
| IX | R.I.F. | — | — | 0·2 ml. 0·025% BP × 2 weekly | 18 | 13 | 37 | 12 | 29 |
| X | P.R.M. + Yeast* | — | — | (as above) | 20 | 12 | 42 | 11 | 28 |
| XI | R.I.F. | Thiamine 0·2% | Thiamine 2·5 mg. × 2 weekly† | (as above) | 8 | 5 | 12 | 5 | 8 |
| XII | R.I.F. | — | Riboflavin 0·25 mg. × 2 weekly | (as above) | 19 | 8 | 13 | 8 | 21 |

* 2 oz. per cage of 10 mice twice weekly.

† Stopped after 20 weeks.

the two DMBA-treated groups. Among the BP-treated groups the incidence was slightly, but not significantly, lower in Groups XI and XII than in Groups IX and X.

Apart from those of Group XI, mice in all groups thrived and gained weight at approximately the same rate. Those in Group XI were underweight when the injections of thiamine were stopped at the 20th week, but thereafter picked up, their average weights approaching those in other groups by the 33rd week.

It was concluded that neither the differences in diet not the administration of thiamine or riboflavin affected skin carcinogenesis by repeated applications of DMBA ; but that injected riboflavin may have slightly reduced the incidence of papillomas at 20 weeks, and of malignant tumours arising before the 33rd week, in mice treated repeatedly with BP.

### *Experiment III*

After randomization 3 groups each of 20 male and 20 female mice began treatment with the diets shown in Table III. Four weeks later all groups began twice weekly applications to the skin of 0·2 ml. 0·025 per cent BP in acetone.

Papillomas began to appear in Groups XIII and XIV during the 11th week of treatment, and in Group XV two weeks later. During the next few weeks Group XV lagged behind the other two in tumour development, but from the 18th week onwards this difference progressively disappeared. Table III shows that by the 23rd week there was very little difference between the three groups. The experiment was therefore abandoned at this point.

It is perhaps of passing interest that the mice of Group XV appeared healthier and were always on the average slightly heavier than mice in the other two groups throughout the experiment. Survival in this group was also much better. However, it is well known that intercurrent infections frequently affect one cage of mice more than another. Also it should be noted that Group XIV was never intermediate between the other two groups in health, weight, or survival. Therefore the apparent beneficial effect of riboflavin in Group XV requires confirmation.

TABLE III.—*Effect of Dietary Riboflavin-level on Induction of Skin Tumours by Repeated Applications of BP*

| Group | Diet | Applications to skin (begun 4 weeks after start of special diets) | Mice in group | Tumour incidence after 23 weeks: Survivors | Mice with tumours | Total benign tumours | Total malignant tumours | Average weight gain during experiment (g.) |
|---|---|---|---|---|---|---|---|---|
| XII | 41B | 0·2 ml. 0·025% BP in acetone twice weekly for 23 weeks | 40 | 30 | 21 | 76 | 6 | 8·0 |
| XIV | 41B + 0·2% Riboflavin | | 40 | 28 | 25 | 74 | 6 | 7·2 |
| XV | 41B + 0·6% Riboflavin | | 38 | 37 | 32 | 71 | 6 | 8·2 |

### DISCUSSION

One hesitates to report experiments that give negative or inconclusive results. However, in the field of carcinogenesis there is a tendency to invoke special factors in the diet as being responsible for unexpected results. This was the reason for undertaking the experiments reported, and by reporting them it is hoped that others will be dissuaded from setting up similar experiments. The inclusion of 0·6 per cent riboflavin in the diet of mice, equivalent to more than 150 mg. per mouse per day, in Group XV (Experiment III) is surely a fairly stringent test of the ability of riboflavin to inhibit carcinogenesis by repeated applications of 3,4-benzopyrene to the skin.

The mystery referred to in the introduction of the failure of Strong A mice to develop tumours in response to applications of benzopyrene remains unsolved. It may have had nothing to do with diet. Alternatively, it may have been due to a factor present in one batch of P.R.M. diet but not in others. The latter explanation is supported by the fact that later batches were apparently less yellow than earlier ones. However, detailed enquiries into the possible differences between batches have consistently failed to provide a basis for planning further critical experiments.

Tannenbaum and Silverstone (1953) with respect to the effect of vitamins in the diet on carcinogenesis concluded :

" Analysis of the findings on the influence of varying the level of B vitamins suggests that wide changes in dietary content, in the range above minimal needs, have little effect on carcinogenesis. At least, this appears to be valid for the induced skin tumour and the spontaneous mammary, lung and liver tumours of the mouse. As deficiency levels are approached there may be some inhibition of carcinogenesis, but in these instances caloric intake and body weight changes may be the major cause of the altered response."

This conclusion is not changed by the results of the experiments reported here.

### SUMMARY

The administration of massive doses of riboflavin to " 101 " strain mice had no more than a slight inhibitory effect on their development of skin tumours in response to repeated applications of 3,4-benzopyrene. Additions of other vitamins of the B group had no definite effect at all.

This work was supported by a block grant from the British Empire Cancer Campaign for which I am greatly indebted. Gratitude is also due to Dr. G. Calcutt, Mount Vernon Hospital, Northwood, Middlesex, for advice and provision of certain materials, and to Miss Celia de Mengel and other members of the staff of the Cancer Research Department, London Hospital Medical College, for assistance.

### REFERENCES

BOUTWELL, R. K., BRUSH, M. K. AND RUSCH, P.—(1949) *Cancer Res.*, **9**, 747.
BRUCE, H. M.—(1950) *J. Hyg., Camb.*, **48**, 171.
*Idem* AND PARKES, A. S.—(1949) *Ibid.*, **47**, 202.
TANNENBAUM, A. AND SILVERSTONE, H.—(1953) *Advanc. Cancer Res.*, **1**, 451.
THOMSON, W.—(1936) *J. Hyg. Camb.*, **36**, 24.